# Research on a Pulmonary Nodule Segmentation Method Combining Fast Self-Adaptive FCM and Classification

**DOI:** 10.1155/2015/185726

**Published:** 2015-04-07

**Authors:** Hui Liu, Cai-Ming Zhang, Zhi-Yuan Su, Kai Wang, Kai Deng

**Affiliations:** ^1^School of Computer Science and Technology, Shandong University of Finance and Economics, Jinan 250014, China; ^2^Digital Media Technology Key Lab of Shandong Province, Jinan 250014, China; ^3^Lawrence Berkeley National Lab, University of California, Berkeley, CA 94720, USA; ^4^Respiratory Department, Shandong Provincial Qianfoshan Hospital, Jinan 250014, China

## Abstract

The key problem of computer-aided diagnosis (CAD) of lung cancer is to segment pathologically changed tissues fast and accurately. As pulmonary nodules are potential manifestation of lung cancer, we propose a fast and self-adaptive pulmonary nodules segmentation method based on a combination of FCM clustering and classification learning. The enhanced spatial function considers contributions to fuzzy membership from both the grayscale similarity between central pixels and single neighboring pixels and the spatial similarity between central pixels and neighborhood and improves effectively the convergence rate and self-adaptivity of the algorithm. Experimental results show that the proposed method can achieve more accurate segmentation of vascular adhesion, pleural adhesion, and ground glass opacity (GGO) pulmonary nodules than other typical algorithms.

## 1. Introduction

In recent years, morbidity and mortality rates of lung diseases in China are growing annually, resulting from environmental degradation induced by air pollution and increase of smoking and passive smoking. As indicated by data provided by National Cancer Institute, Ministry of Health, the mortality rate of lung cancer in China increased by 465% and its morbidity rate increased by 26.9% in the past 30 years, and furthermore, rather than liver cancer, lung cancer has been the prime cause of death due to malignant tumors, which takes up 22.7% of all such deaths (morbidity and mortality due to lung cancer increase drastically, http://health.sohu.com/20121127/n358789664.shtml). Therefore, research of computer-aided diagnosis on lung cancer has being increasingly important.

Currently, as far as the characterization of lung diseases is concerned, CT imaging is the best imageological means to diagnose lung diseases among various medical imaging types [1]. Since pulmonary nodules are the most commonly seen symptoms in the early stage of lung cancer, automatic extraction and assisted diagnosis of pulmonary nodules by means of spiral CT play a significant role in the detection of early lung cancer [2], which reduces effectively the errors in interpreting images by eyeballs and the work load of image reading. More importantly, it improves the repeatability of diagnosis and consistency of image interpretation to some extent by providing accurate quantitative analysis for doctors by techniques such as image segmentation and quantification of characteristics [3].

In the process of research, a series of characteristics of pulmonary nodules such as size, form, density, and patterns of strengthening and growth need to be analyzed comprehensively in order to provide a basis for identification of benignity and malignancy of tumors [4]. For instance, pulmonary nodules have clear edges and the form of edges (smooth, lobulated, speculated protuberance, or burr) are key indicators of benignity and malignancy of tumors. So it cannot be more important to implement correct segmentations of pulmonary nodules.

**Figure 1:**
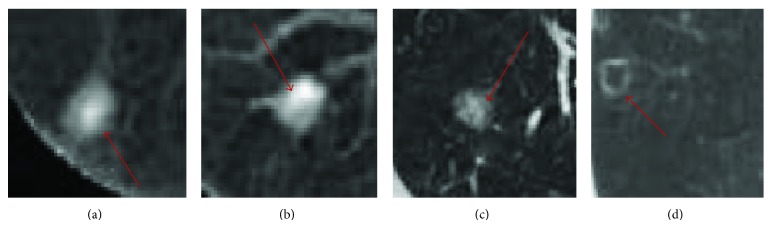
All kinds of pulmonary nodules: (a) vascular adhesion, (b) pleural adhesion, (c) ground glass opacity, and (d) cavitary nodule.

In recent years, a lot of pulmonary nodule segmentations methods have been proposed, which can be categorized as threshold methods [5, 6], morphological methods [7], variable model methods [8], clustering methods [9–11], and image segmentation methods [12, 13]. Nevertheless, many contemporary methods need human interventions and most of them perform well only for isolated solid pulmonary nodules. However, pulmonary nodules usually occur in forms of vascular adhesion, pleural adhesion, ground glass opacity (GGO), and cavitary pulmonary nodules, as demonstrated in Figure 1. These types of nodules have higher probability of malignancy than isolated ones. Obviously, because of attachment of these nodules to surrounding tissues, fuzzy edges, and similar gray scales, fast, correct, and automatic segmentation of these nodules is still a difficult problem.

Fuzzy *C*-means (FCM) algorithm [14] is a popular algorithm and has been widely used in image segmentation; it applies fuzziness to the membership judgment of pixels, which is consistent with human perception and convenient for realization. However, conventional FCM algorithm does not consider any spatial information and neighborhood correlation, which makes it perform poor in low contrast, in-homogeneity, and noisy images.

In order to overcome these problems, many researchers have introduced spatial information through modifying the objective function or altering the distance form measurement between pixels and cluster centers. Fergus et al. proposed a modified FCM algorithm (SFCMpq), which considers the fact that the adjacent pixels will have similar characteristic value and the probability that they belong to the same class is very big. Chuang et al. [15] proposed a weighted FCM integrating the spatial neighborhood information. Ahmed et al. [16] proposed FCM_S algorithm where the objective function is modified by introducing a neigborhood term, which is used to compensate the intensity inhomogeneity and allow the labeling of a pixel to be influenced by the labels of its immediate neighborhood. However, the execution efficiency of FCM_S is very low [17], since it needs to compute a series of neighborhood labelings during each iteration. Two improved variants, FCM_S1 and FCM_S2, of FCM_S were proposed with an intent to simplify the computation of parameters and then Chen and Zhang [18] extend them to the corresponding kernelized versions, KFCM_S1 and KFCM_S2, by the kernel function substitution. An enhanced FCM (EnFCM) algorithm [19] was proposed to speed up the clustering process on the basis of the gray level histogram instead of pixels, and it uses a linearly weighted sum image formed from both original image and each pixel's local neighborhood average gray level. Hence, the computational time of EnFCM algorithm is reduced greatly since the number of gray levels in an image is much smaller than that of pixels. Fast generalized FCM (FGFCM) algorithm [20] introduces the spatial information combining the intensity of the local pixel neighborhood and the number of gray levels in an image. The quality of segmentation result is well enhanced, and the computational time of FGFCM algorithm is small similarly. Krinidis and Chatzis proposed a novel robust fuzzy local information *C*-means clustering (FLICM) algorithm [21] by incorporating the local spatial and gray level information, which is free of the empirically adjusted parameters and enhances the clustering performance.

Although the above-modified FCM algorithms have improved the performance of conventional FCM and segmented different types of images to some extent, they still have the following disadvantages: (1) they require a large number of iterations to achieve the convergence; (2) they have the worst performance in segmenting CT images of lungs with complicated features; for example, the blood vessels have similar grayscales to pulmonary nodules, GGO pulmonary nodules have low contrast to backgrounds, and so forth.

Discussing these issues, this paper presents a pulmonary nodule segmentation method combining fast self-adaptive FCM and classification. The improved spatial function will calculate the cluster centers adaptive by both grayscale similarity and spatial similarity of pixels neighborhood space and update the fuzzy membership degree of center pixel during each iteration, obtaining the cluster centers at a faster rate. Afterward, we can construct the classifier based on the clustering results according to the labeled information.

## 2. Modified Fast Self-Adaptive FCM Segmentation

In addition to lung tissues, a typical CT image of chest section includes other organs, skeletons, and trachea. To increase the accuracy of CAD detection to lesion tissues and image processing speed, the primary mission of this paper is to remove these irrelevant items in images and extract images of pulmonary parenchyma by means of global binarization of threshold, detecting boundaries of trunks, removing background noises, and inpainting lung areas. Thereafter, nodules in lungs as well as blood vessels and bronchi which have similar characteristics with pulmonary nodules can be analyzed. CT images of pulmonary nodules are generally bright in center and dark in peripheral area so that points mapped into the joint characteristic space of brightness and greyscales are dense in center and scattered in peripheral. Considering the diversity of types of pulmonary nodule density distribution, weak edge distribution of peripheral tissues, and robustness of algorithm to noise, the following modifications of traditional FCM algorithm are proposed in this paper: the new spatial function considers the contributions to fuzzy membership from both the grayscale similarity between central pixels and single neighboring pixels and the spatial similarity between central pixels and neighborhood. The modified algorithm improves effectively the convergence rate and adaptivity to spatial characteristics of pulmonary nodule edges.

### 2.1. The Traditional FCM Algorithm

As a clustering algorithm based on division, the traditional FCM algorithm divides *N* pixels into *C* fuzzy groups, searches the clustering center of each group, and updates the clustering centers through updating fuzzy membership of the pixels relative to each clustering. The update is implemented by means of minimizing the objective function, which is defined as

(1) $$\begin{array}{c} E=\overset{C}{\underset{i=1}{\sum }}\;\overset{N}{\underset{j=1}{\sum }}\mu_{m}^{ij}{\left\|x_{j}-v_{i}\right\|}^{2}, \end{array}$$

where *C* is the amount of clusters, *N* is the amount of pixels in the image, *μ*_*m*_^*ij*^ is the fuzzy membership of the *j*th pixel with respect to the *i*th pixel, *m* is the weight exponent acting on the fuzzy membership, and *v*_*i*_ is the center of the *i*th clustering. The constraint for the objective function minimization is

(2) $$\begin{array}{c} \mu_{m}^{ij}\in \left[0,1\right],\;\overset{C}{\underset{i=1}{\sum }}\mu_{ij}=1. \end{array}$$

Differentiating equation (1) with respect to *μ*_*m*_^*ij*^ and *v*_*i*_ obeying constraint (2) yields yields a necessary condition of minimizing (1):

(3) $$\begin{array}{c} \mu_{m}^{ij}=\frac{1}{\sum_{i=1}^{C}{\left[\left\|x_{j}-v_{i}\right\|/\left\|x_{j}-v_{k}\right\|\right]}^{2/\left(m-1\right)}}, \end{array}$$

(4) $$\begin{array}{c} v_{i}=\frac{\sum_{j=1}^{n}\mu_{m}^{ij}x_{j}}{\sum_{j=1}^{n}\mu_{m}^{ij}}. \end{array}$$

This algorithm minimizes the objective function by assigning higher fuzzy membership to pixels whose greyscales are close to those of specific clustering centers and assigning lower fuzzy membership to pixels whose greyscales are distinct from those of specific clustering centers.

Nonetheless, the traditional FCM algorithm calculates the fuzzy membership of each pixel based on the distance of the pixel and clustering centers, resulting in the fact that it is not suitable to pulmonary nodule images in which characteristics of forms and distributions are complex and diverse because it is difficult to obtain smooth segmentation edges and closed segmentation intervals. In addition, the executive efficiency of the FCM algorithm is low since it needs multiple iterations to converge. Existing modified FCM algorithms, such as FCM_S, FCM_S1, FCM_S2, FGFCM, and EnFCM, take into account information of pixels in neighborhoods of central pixels and introduce a parameter *α* or *λ*_*g*_ that controls the intensity of neighborhood information. This parameter is important because its selection influences directly the performance of algorithm. However, this parameter cannot vary self-adaptively; that is, its value maintains unchanged during the whole process of segmentation for an image. Obviously, the selection of *α* does not reflect the characteristics of the neighborhood of the pixels.

We acclaim that the value of *α* should be chosen based on (1) similarity of local greyscales; that is, *α* should depend on the difference between the greyscales of central pixels and neighboring pixels in order to suppress noise and speed convergence; (2) local spatial similarity; that is, *α* also depends on the size of neighborhoods and the difference between greyscales of neighborhood average and central pixels so that the adaptivity of algorithm is enhanced.

### 2.2. 2D Vector Representation of the Spatial Relationship between a Pixel and Its Neighborhood

For an extracted pulmonary parenchyma image, the amount of pixels is denoted as *N* and greyscale lies in the interval [0, *L* − 1], the neighborhood of a pixel *x*_*j*_, *j* ∈ [0, *N*], is *N*_*r*_, and *x*_*r*_ is a pixel in the neighborhood. Denote the greyscale at the central pixel *x*_*j*_ as *f*(*x*_*j*_)∈[*x*, *L* − 1] and the average value in a neighborhood centered at this pixel with the size (2*r* + 1)×(2*r* + 1) as *g*(*x*_*j*_), where the parameter *r* is determined based on image resolution and noise. The 2-dimensional vector [*f*(*x*_*i*_), *g*(*x*_*j*_)] represents the greyscale at *x*_*j*_ and that of its neighborhood; it will have *M* ^*^ different values in the whole lung parenchyma. The algorithm segments the image by sampling the above 2D vector instead of the traditional greyscale.

To analyze the statistical rules of the correspondence between 2D vectors and various tissues in pulmonary parenchyma, a 2D greyscale matrix is defined and constructed, in which the abscissa represents the greyscales at pixels and the ordinate represents the average in their neighborhoods. The frequency *p*_*j*_ of the vector value [*f*(*x*_*j*_), *g*(*x*_*j*_)] is

(5) $$\begin{array}{c} p_{j}=\frac{n_{j}}{N},\;\overset{M^{*}}{\underset{j=1}{\sum }}p_{j}=1, \end{array}$$

where *n*_*j*_ means the number of times that the vector value [*f*(*x*_*j*_), *g*(*x*_*j*_)] occurs.

**Figure 2:**
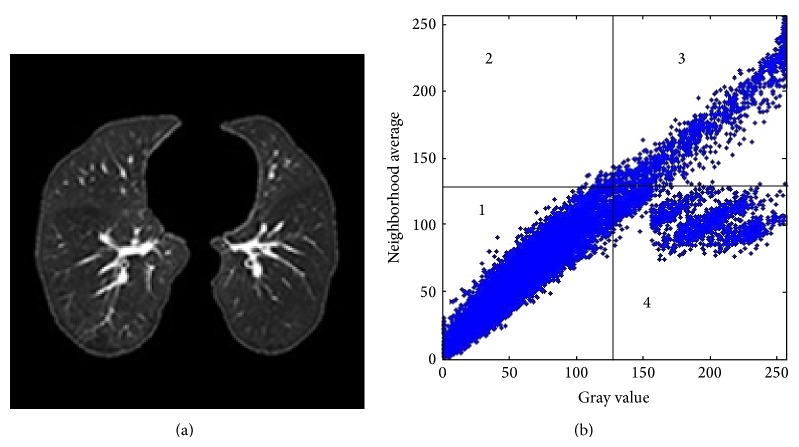
Greyscale and neighborhood average: (a) a pulmonary parenchyma image and (b) 2D vector distribution; regions 1 and 3 correspond to the smooth area, region 4 corresponds to the edges of nodules, tracheas, great blood vessels, and so forth.

Figure 2(b) shows the distribution of greyscales and neighborhood averages of pixels corresponding to pulmonary parenchyma shown in Figure 2(a), for which *r* is chosen as 2. By statistical analysis from the 2D vector distributions at all pixels, since pulmonary parenchyma is the background in images, the greyscale values at corresponding pixels are close to their neighborhood averages, which is indicated by points around the diagonal in region 1 in Figure 2(b); greyscale values at pixels inside tracheas, blood vessels, and pulmonary nodules are also close to their neighborhood averages, indicated by points around the diagonal in region 3 in Figure 2(b); amount of pixels at edges of nodules, tracheas, and great blood vessels is relatively large and greyscales at these areas are significantly different from neighborhood averages, shown by points densely distributed in region 4. In addition, discrete points showing low frequency of occurrence and huge difference between local greyscale values and neighborhood averages correspond to pixels at tiny blood vessel edges and lung wall edges.

From above, for a pixel, the size of its neighborhood, the difference between local greyscale and neighborhood average, and its frequency *p* can provide local pattern features of the neighborhood around the central pixel for the algorithm updating fuzzy membership.

### 2.3. FCM Algorithm Based on Weighted Spatial Information and Greyscale Information

To improve the traditional FCM algorithm, this paper introduces a new spatial function which includes the spatial features and greyscale information of the neighborhood around the central pixel. The spatial function is defined as

(6) $$\begin{array}{c} h_{ij}=\underset{x_{r}\in N_{r}}{\sum }\mu_{ir}+\omega_{r}, \end{array}$$

where *h*_*ij*_ represents the probability that the pixel determined from spatial information of the neighborhood belongs the *i*th kind and *μ*_*ir*_ is the membership that the neighboring pixels *x*_*r*_ belong to the *i*th kind, which is calculated by (3). Obviously, *h*_*ij*_ is maximum if all pixels in the neighborhood of the pixel *x*_*j*_ belong to the *i*th kind. Otherwise, *h*_*ij*_ is minimum if all pixels in the neighborhood of the pixel do not belong to the *i*th kind.

A polynomial *ω*_*r*_ represents the global scale factor determined by the difference between greyscales at the pixel *x*_*j*_ and neighboring pixels *x*_*i*_; that is, the spatial function *h*_*ij*_ depends on the fuzzy membership of neighboring pixels *x*_*r*_, and more importantly contributions to the spatial function *h*_*ij*_ from different neighboring pixels *x*_*r*_ around *x*_*j*_ should be distinct. The less the greyscales at *x*_*j*_ and *x*_*r*_ are different, the closer their relation is and the greater the corresponding weight coefficient should be. Otherwise, the weight coefficient should be smaller. Thus, *ω*_*r*_ is defined as

(7) $$\begin{array}{c} \omega_{r}=\frac{1}{\exp\left({\left|x_{j}-x_{r}\right|}^{2}\right)\cdot \lambda_{j}}, \end{array}$$

where *λ*_*j*_ is equivalent to the parameter *α* in other classical algorithms. But *λ*_*j*_ is able to adjust the difference between greyscales at central pixels and their neighborhoods according to the degree of importance of the 2D vector in local pattern space. Consider

(8) $$\begin{array}{c} \lambda_{j}=p_{j}\cdot \sqrt{\underset{x_{r}\in N_{r}}{\sum }\frac{{\left(f\left(x_{r}\right)-g\left(x_{j}\right)\right)}^{2}}{{\left(2r+1\right)}^{2}}} \end{array}$$

which shows that *λ*_*j*_ is variable and enhances the self-adaptivity of pixels to their neighborhoods.

It can be seen that formula (7) combines the greyscales at central pixels and pixels in their neighborhoods and adjust dynamically the brightness around central pixels depending on the sizes of neighborhoods and mean square deviation in neighborhoods. This practice protects the pixels in smooth areas, magnifies the differences in edges, and eliminates noise and edges of tiny suspect objects in the fuzzy membership matrix.

The spatial function *h*_*ij*_ is plugged into the following formula in order to update the fuzzy membership of the pixel *x*_*j*_:

(9) $$\begin{array}{c} \mu_{ij}^{\prime }=\frac{\mu_{ij}^{p}\cdot h_{ij}^{q}}{\sum_{k=1}^{C}\mu_{ij}^{p}\cdot h_{ij}^{q}}, \end{array}$$

where parameters *p* and *q* control the relative significance of the two functions.

Based on the above descriptions, the self-adaptive FCM algorithm for training is as follows:

**Algorithm 1 (fast self-adaptive FCM).**

Consider the following.

*Input*. Extract CT image of pulmonary parenchyma.

*Output*. Segment pulmonary nodules.

*Initializations*. Choose the amount of clustering centers *C* = 2, the maximum number of iterations *L*_max⁡_ = 100, fuzzy weight exponent *m* = 2, size parameter of neighborhoods *r* = 2, parameters that control the relative significance of the two functions *p* = 1 and *q* = 2, and termination threshold *ε* = 0.001; set counter for iteration *l*0; initialize randomly a clustering prototype matrix *V* ^(0)^ and normalize this matrix so that it satisfies constraint (2); finally, obtain clustering centers *v*_*i*_^(0)^ using formulas (3) and (4).

*Step 1*. Calculate and plot the 2D histogram for the pulmonary parenchyma image and obtain frequencies of occurrence *p*_*j*_ of all 2D vectors.

*Step 2*. Calculate fuzzy membership *μ*_*ij*_^*l*^ of pixel *x*_*j*_ and assemble the partition matrix *U* ^(*l*)^:

(10) ∀i,j,  ∃xj−vil>0,  thenμijl=∑k=1Cxj−vilxj−vkl2/m−1−1.

If ∃*i*, *r*, such that |*x*_*j*_ − *v*_*i*_|^(*l*)^ = 0, then *μ*_*ir*_^(*l*)^ = 1 and (*μ*_*ir*_^(*l*)^ = 0 for *i* ≠ *k*).

*Step 3*. Update fuzzy memberships according to formulae (7)–(9) and obtain new fuzzy memberships *μ*_*ij*_^′(*l*)^.

*Step 4*. Update clustering prototype modes *V* ^(*l*)^; that is, update clustering centers *v*_*i*_^(*l*+1)^ using new memberships *μ*_*ij*_^′(*l*)^:

(11) vil+1=∑j=1Nμij′lmxj+1/NR∑xr∈NRxr∑j=1Nμij′lm,i=1,2,…,C.

*Step 5*. If ‖*V* ^(*l*+1)^ − *V* ^(*l*)^‖ < *ε* or the maximum count of iteration *L*_max⁡_ is reached, enter Step* *6; otherwise, return to Step* *3.

*Step 6*. Deblur using the method of maximum membership function. The type of clustering that the pixel *x*_*j*_ belongs to is represented by *c*_*j*_:

(12) $$\begin{array}{c} c_{j}=\underset{i}{\arg}\left\{\max\left(\mu_{ij}\right)\right\}\;\forall i,\;\;\forall k. \end{array}$$

In the algorithm, the fuzzy weight exponent *m* is a parameter introduced to generalize fuzzy clustering objective functions by Bezdek, which plays a role in suppressing noise and smoothing membership functions. The empirical range of *m* given by Bezdek is 1.1 ≤ *m* ≤ 5. Later, the most suitable physical interpretation was obtained for *m* = 2 [22]. Thus *m* is chosen as 2 in this paper.

Now, let us analyze the number of iterations and running time of the algorithm for image processing. Since the iterations loop over each pixel, the time complexity of the algorithm depends closely on amount of pixels and number of iterations, that is, *O*(*NcL*), where *N* is the amount of pixels, *c* is the number of clusters, and *L* is the number of iterations. The operational speed of the algorithm in this paper is significantly improved because the number of iterations of this algorithm is much less than traditional FCM and FCM_S algorithms. Besides, EnFCM and FGFCM algorithms transform segmentation based on pixels into segmentation based on greyscale (0–255) levels. Due to the fact that the number of greyscale levels is much less than amount of pixels, the speed of algorithm becomes much faster.

**Figure 3:**
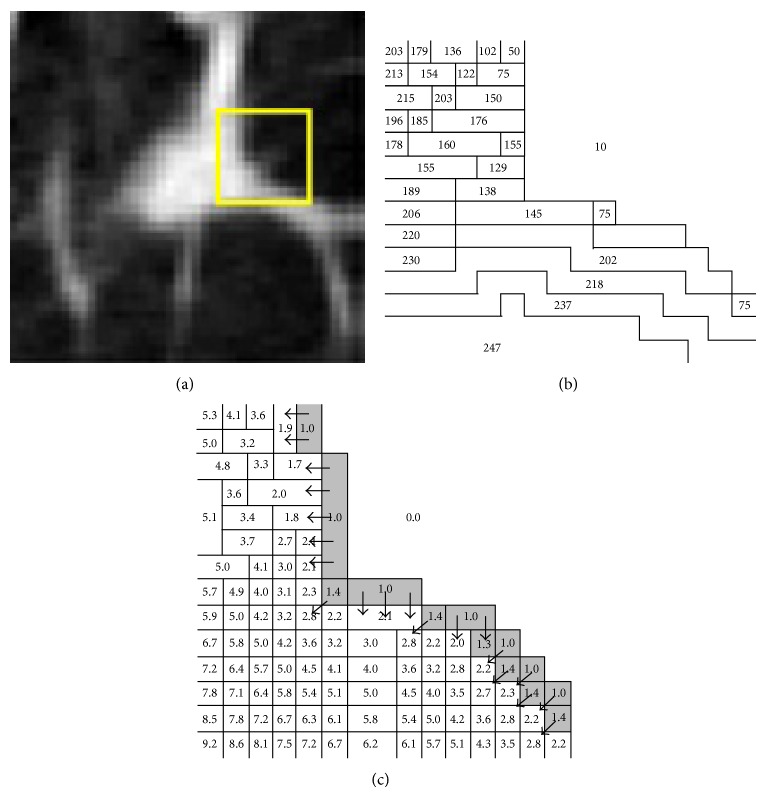
Greyscale map and memberships at pixels in local segmentation area of blood vessel adhesion nodules: (a) pulmonary nodules to be segmented, (b) distribution of grayscales, and (c) the membership change trend of boundary pixels.

Figure 3 demonstrates the results of fuzzy memberships of pixels at pulmonary parenchyma, edges, and pulmonary nodules given by the above algorithm applied to an image of blood vessel adhesion pulmonary nodules. The yellow rectangular area in Figure 3(a) is the area to be analyzed.  Figure 3(b) shows the distribution of greyscales at pixels in the rectangular area in the form of level sets.  Figure 3(c) indicates the results of fuzzy memberships at the pixels in the interesting area relative to the foreground clustering *c*_*F*_ calculated by using the new spatial function. To make the demonstration of results more visually sense-making, the values shown in the figure are the results that actual values are multiplied by 100 and rounded. Black arrows point to the evolutionary directions of fuzzy memberships. Pixels in shadow areas represent segmented boundaries based on the membership threshold.

**Figure 4:**
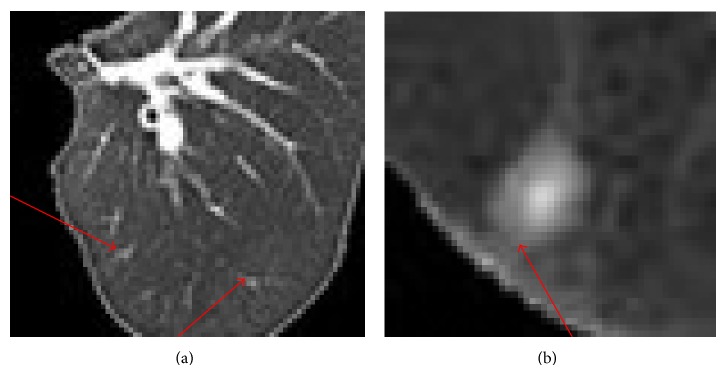
Example to be analyzed: (a) noise of tiny blood vessels; (b) connection between nodule and pleura.

Let us elaborate the effectiveness of the new spatial function by taking the pulmonary parenchyma shown in Figure 4, for instance. The positions that arrows point to can be regarded as noise. The frequency *p* that the corresponding 2D vector occurs is extremely small and its greyscale differs significantly from most neighboring pixels. Thereafter, in the initial stage, *μ*_*ij*_ is relatively small while *μ*_*ir*_ is large, which lead to the result that *h*_*ij*_ given by the algorithm through the spatial function (6) is large and the new fuzzy memberships *μ*_*ij*_′ by substituting *h*_*ij*_ into formula (9) is also relatively large. After each iteration, the probability that this pixel enters the *i*th clustering increases until the termination of the algorithm. Thus, this pixel will be finally categorized into the *i*th clustering where its neighboring pixels belong to, although its greyscale is distinct from the center of the *i*th clustering *v*_*i*_. It is right opposite to the case of pleural adhesion nodules shown in Figure 4(b), in which the area of pulmonary nodules extends until being connected to pleura that shows higher greyscale levels. Though greyscale levels at the connections are low, they transit gradually so that the difference of greyscales at central pixels and neighborhoods is relatively small. The factor *λ*_*j*_ in formula (8) adjusts self-adaptively the brightness around pixels in the connection area by increasing the weight of *μ*_*ij*_, which also increases the spatial function *h*_*ij*_. After one iteration, fuzzy membership changes significantly.

1. The central pixel is a point of noise and pixels in its neighborhood are homogeneous in greyscale. Let us take a point of tiny blood vessel noise shown in Figure 4(a) as an example, in which the size parameter of neighborhood *r* = 1. Greyscales and initial fuzzy memberships of the pixels in this area are listed in Table 1(a). Tables 2(a), 2(b), and 2(c) demonstrate the memberships *μ*_*ij*_′ and clustering centers *v*_*i*_ of the pixels in this area after 1, 4, and 7 iterations. In Table 2, the upper and lower parts show results given by formula (1) and Step 3 in Algorithm 1, respectively. By comparing the memberships and clustering centers before and after updating, it can be seen that clustering centers after each iteration are rarely influenced by central pixels, speed of convergence is improved significantly, and fuzzy memberships of pixels in neighborhoods tend to be consistent. Thus the noise of tiny blood vessels is correctly categorized into the background clustering *c*_*B*_.
2. The central pixel is not a point of noise but is homogeneous in greyscale with pixels in its neighborhood.  Table 3 demonstrates the memberships and clustering centers of the pixels in the neighborhood of the example pixel shown in Table 1(b) after 1, 4, and 7 iterations. It can be seen that since the central pixel of the neighborhood is not noise, only the greyscales at diagonal pixels are relatively dark and the greyscales in the neighborhood show smooth transitions. Through comparing the memberships *μ*_*ij*_′ and clustering centers *v*_*i*_ before and after updating as listed in Tables 3(a), 3(b), and 3(c), it is observed that after one iteration in the algorithm proposed by this paper, memberships *μ*_*ij*_′ at pixels in the neighborhood are lower than those computed by Step 2 of Algorithm 1 and membership values tend to be consistent after several iterations. The central pixel is correctly categorized into the foreground clustering *c*_*F*_.

The above two examples provide theoretical explanations about the robustness of the algorithm. From the point of view of localized methods, formula (6) fully combines the local spatial information and greyscales of the image, which enhances the robustness of the algorithm to noise or outliers and the self-adaptivity to homogenous neighborhoods. In the meantime, it can change the fuzzy memberships of central pixels significantly and improve the executive efficiency of the algorithm.

## 3. Experimental Results and Analysis

This paper uses experimental data from whole lung image series provided by the Lung Image Database Consortium (LIDC (USA Lung Image Database Consortium (LIDC), https://public.cancerimagingarchive.net/ncia/login.jsf)) [23], USA, and the lung CT image database of Qianfoshan Hospital, Shandong Province.  Table 4 describes the two sets of experimental data, 2D CT images selected for experiments, and information about pulmonary nodules. The experimental images contain labels of pulmonary nodules provided by clinical experts.

To verify the effectiveness of the newly proposed algorithm, the new algorithm is compared with 5 existing typical algorithms (FCM, FCM_S, EnFCM, FGFCM, and FLICM) about effect of segmentation, operational efficiency, and error rate. In the whole experiment, parameters involved in the above 5 algorithms are as follows: *m* = 2, *α* = 0.85, *ε* = 0.001, *λ*_*g*_ = 6, and *N*_*R*_ = 9; that is, local neighborhoods are 3 × 3 in size.

### 3.1. Effect of Pulmonary Nodule Segmentations

Segmentations to isolated, blood vessel adhesion, pleural adhesion, and ground glass opacity pulmonary nodules are implemented and compared in order to verify and compare the performance of the above algorithms on removing noise and treating tiny tissues, objects with fuzzy edges, and objects with similar greyscales.

**Figure 5:**
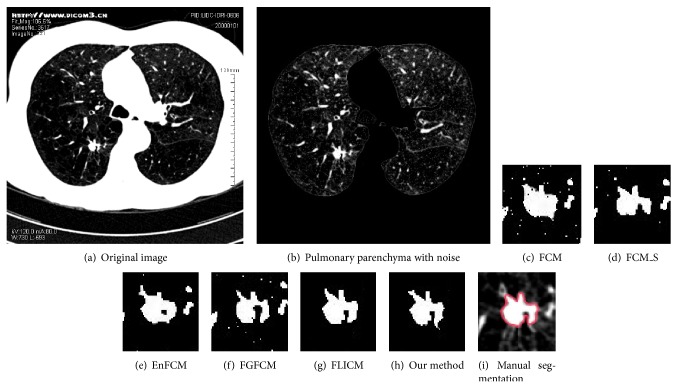
Segmentation results of isolated pulmonary nodules with salt and pepper noise.

Figure 5 demonstrates the image of an isolated pulmonary nodule, the image of pulmonary parenchyma with salt and pepper noise, and images of locally zoomed-in segmentation results obtained by the above six algorithms and by manual segmenting. Through analysis of the segmentation results shown in Figures 5(c)–5(h), it can be found that the performances of FCM and FGFCM are influenced by noise so severely that they cannot separate objectives from background. A large amount of noise still exists in the result of background segmentation and even tiny blood vessels are segmented as objectives. FCM_S and EnFCM perform better than above algorithms, although the segmentation effects of objectives and tiny blood vessels are much less than ideal. The algorithm proposed by this paper can remove the influence of noise satisfactorily and successfully separate objectives from background.

**Figure 6:**
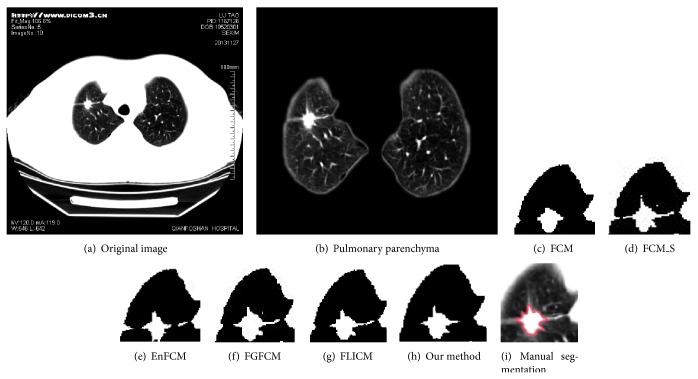
Segmentation results of blood vessel adhesion pulmonary nodules.

Figure 6(a) shows the CT image of a blood vessel adhesion pulmonary nodule. The segmentation results of this nodule by the above six algorithms are demonstrated by Figures 6(b)–6(g). It is observed that, among all these algorithms, FCM yields a result that differs the most from that given by manual segmentation because it only extracts the brightest region in the center of the nodule and loses all information about nodule boundaries. Segmentations provided by FCM_S, EnFCM, FGFCM, and FLICM show errors of boundary leakage at the connections of nodules and blood vessels, and all of these four algorithms make more or less wrong segmentations to left lung walls connected to blood vessels. The method proposed by this paper overcomes these problems and performs well in segmenting blood vessel adhesion pulmonary nodules.

**Figure 7:**
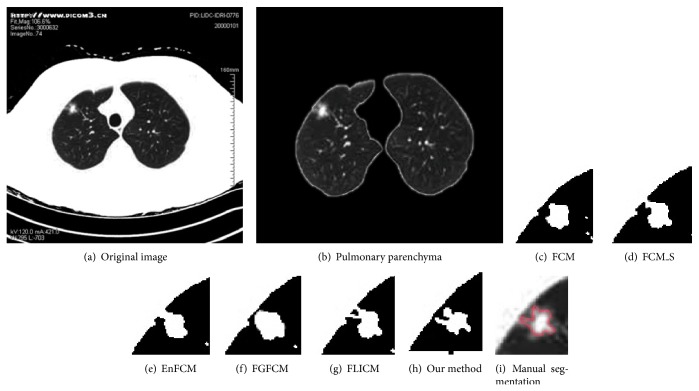
Segmentation results of pleura adhesion pulmonary nodules.

Figure 7(a) shows the CT image of a pleura adhesion pulmonary nodule. The segmentation results of this nodule by the above six algorithms are demonstrated for comparison by Figures 7(b)–7(g). The shape of pulmonary nodule edges segmented by our method are the closest to those provided by experts' manual segmentations. The connections between nodules and pleura are especially delicate, indicated by the fact the extended area of nodules and background areas of pulmonary parenchyma are distinguished.

**Figure 8:**
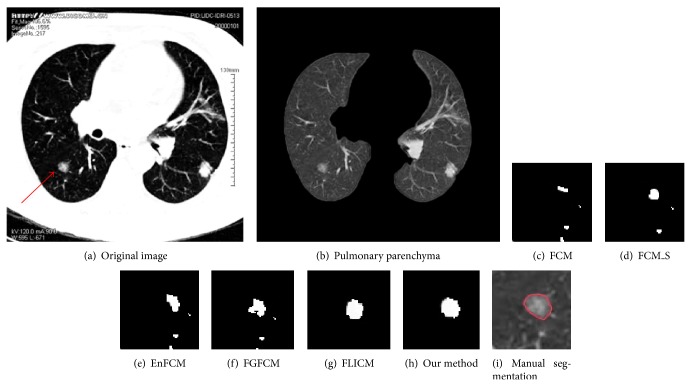
Segmentation results of ground glass opacity pulmonary nodules.

Figure 8(a) shows the CT image of a ground glass opacity pulmonary nodule, which is indicated by a red arrow. The segmentation results of this nodule by the above six algorithms are demonstrated for comparison by Figures 8(b)–8(g). It can be seen that FCM hardly segments pulmonary nodules. FCM_S, EnFCM, and FGFCM perform less than satisfactorily on segmentations of nodules and tiny blood vessels. Segmentations by FLICM are good and our method gives segmentations that are closer to edges expected by experts.

Generally, blood vessels have similar grayscales to pulmonary nodules; GGO pulmonary nodules have low contrast to backgrounds; grayscales at connections between pleura and pulmonary nodules transit smoothly, but the contrast is also very low, compared to the grayscales of backgrounds. The traditional FCM algorithm implements segmentations of images by clustering similar data points in the characteristic space and does not include interactions between neighboring pixels, which yields the worst performance in segmenting CT images of lungs with complicated features. Improvements by introducing neighborhood information and methods to measure distances and correcting memberships are made by all other four typical algorithms, which remove the influence of image noise. However, more or less defects exist in considering spatial similarities and grayscale similarities of pixels in neighborhoods so that these algorithms show problems of falling into local extrema and being sensitive to initial values.

**Figure 9:**
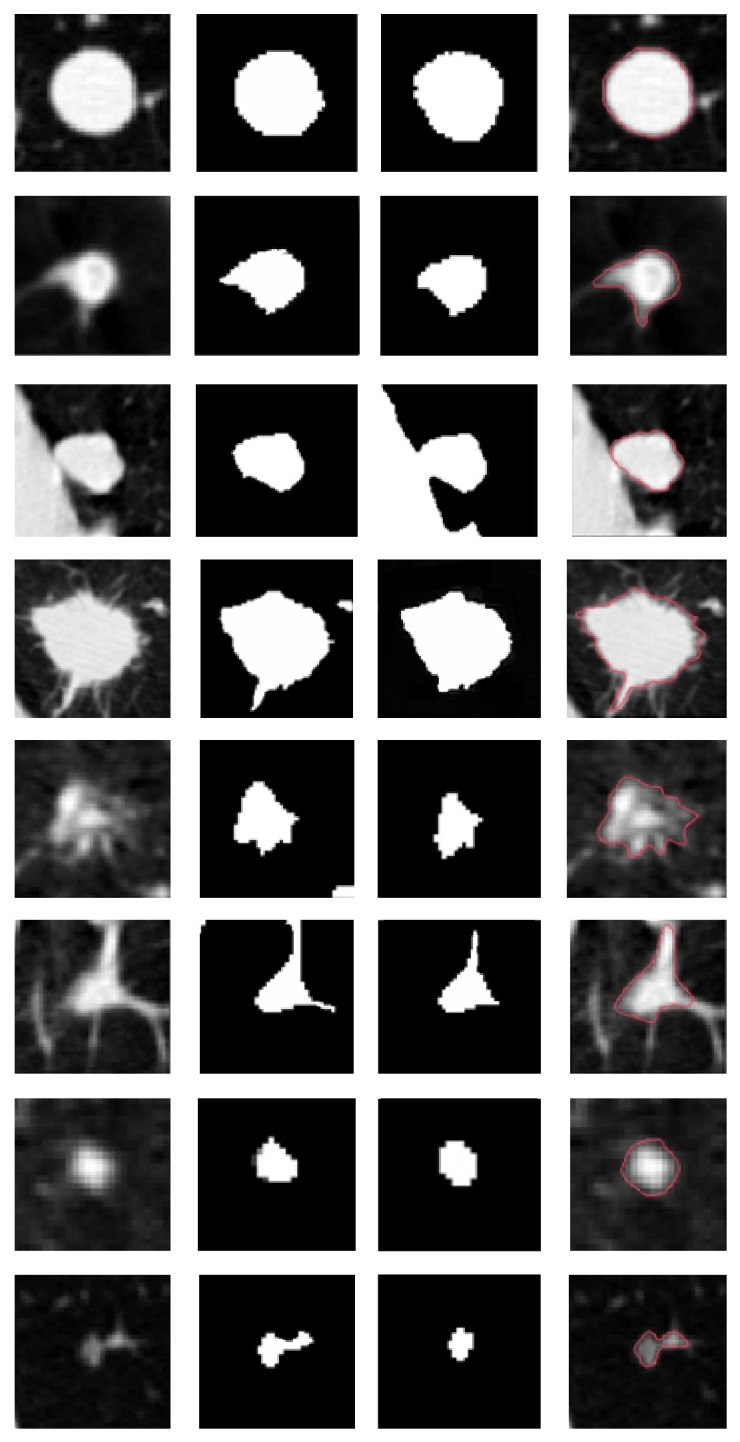
Examples of pulmonary nodule segmentations by our algorithm compared with manual segmentations. For each case, four images are shown. They are, from left to right, the original CT slice, segmentation result with *r* = 1 and *r* = 2, respectively, and manual segmentation result.

To illustrate more results of segmentations of pulmonary nodules, Figure 9 demonstrates several typical pulmonary nodules, including isolated, adhesion, and GGO nodules. Column 1 shows locally zoomed-in images of original pulmonary parenchyma; column 2 shows segmentation results given by using the neighborhood size parameter *r* = 1, that is, using 3 × 3 neighborhoods; column 3 shows segmentation results given by using the neighborhood size parameter *r* = 2, that is, using 5 × 5 neighborhoods; column 4 shows manual segmentation results provided by experts for reference.

Analysis of experimental results demonstrates the following: (1) to some extent, the segmentation obtained by our method is close to the manual references, especially at nodule edges where burrs are obvious. (2) Images in the same group can be referred to each other and those in different groups cannot be compared because different CT images are distinct in doses, resolutions, and quality, especially grayscale, and in the process of implementation the algorithm adjusts the parameter *λ*_*j*_ self-adaptively according to the grayscale features of the current image. (3) Neighborhood size influences the segmentation significantly. Nodule edges which resulted from *r* = 1 are relatively fine, especially where nodules are adhered to blood vessels or lung walls, as shown in the 5th and 6th images; however oversegmentations and wrong segmentations to tiny blood vessels are easy to occur, as shown in the 4th and 5th images. Comparatively, neighborhoods when *r* = 2 are larger and the difference between grayscales at edges and the neighborhood averages increases. Parts of segmentation results are close to manual segmentations, like the 2nd and 6th images. However, undersegmentations occur sometimes, as shown in the 5th and 8th images, and it is also possible to segment nodules that are tightly adhered, as the 3rd image.

### 3.2. Executive Efficiencies of Algorithms

In order to further demonstrate the performance of the algorithm proposed by this paper, Table 5 shows average numbers of iterations (maximum number of iterations *L*_max⁡_ = 100) and running time (seconds) to obtain segmentation results in Figures 5–8 by the above five typical algorithms and our algorithm.

Table 5 indicates that when the FCM algorithm is employed to segment images, the more the iterations are needed to satisfy the convergence threshold value *ε*, the longer the program runs. Other algorithms (FCM_S, EnFCM, FGFCM, and FLICM) can converge with less iterations and have higher efficiencies. The algorithm proposed in this paper needs the least number of iterations and has the shortest running time, which is consistent with the observation described in Section 2.3 that fuzzy memberships change considerably after one iteration. In the meantime, prior knowledge plays a role in supervising unlabeled or sparsely labeled data, which speeds up the convergence of the objective function when improving the effect of clustering.

### 3.3. Error Rate of Segmentation

**Figure 10:**
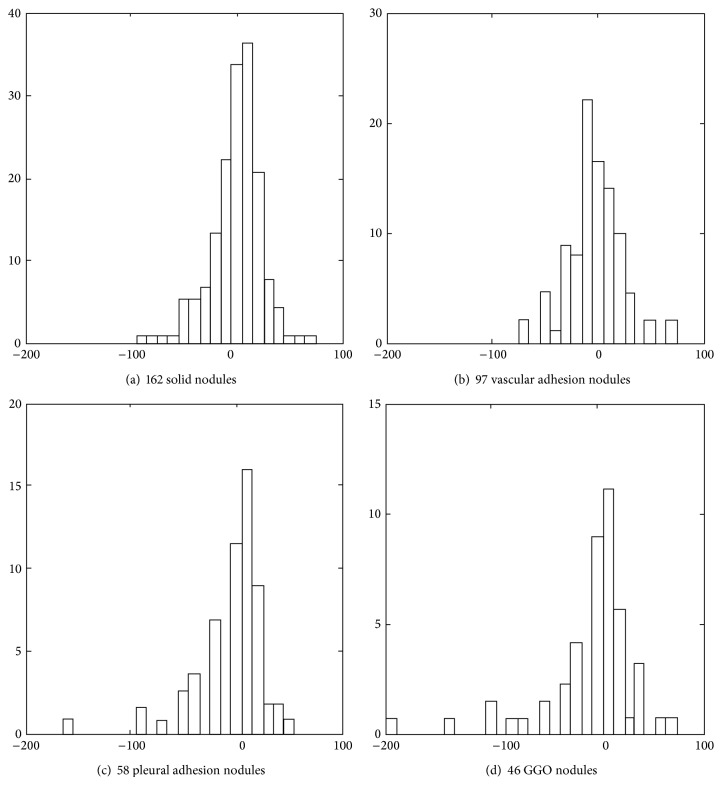
Histogram for comparison between our method and manual segmentation.

Figure 10 demonstrates the comparison between boundaries of all types of pulmonary nodules by our algorithm *e*_*a*_ and those obtained by manual segmentations of experts *e*_*m*_. Figures 10(a), 10(b), 10(c), and 10(d) correspond to four types of pulmonary nodules in test samples with different amounts. In each plot of Figure 10, the ordinate represents number of pulmonary nodules and the abscissa represents rate of wrong segmentations (*e*_*m*_ − *e*_*a*_)/*e*_*m*_ (%). A positive rate of wrong segmentations means undersegmentation resulting from the algorithm with respect to manual segmentations. Otherwise, a negative rate of wrong segmentations means oversegmentation resulting from the algorithm with respect to manual segmentations.

It is seen from Figure 10 that the algorithm performs the worst for GGO nodules. The proportion of GGO nodules whose rates of wrong segmentations lie in the interval [−50%, +50%] is less than 70%. This proportion for any of the other three types of nodules reaches 85%. It is also noticeable that the rates of wrong segmentations for some nodules approach −200 or 100.

## 4. Conclusions

In this paper, a fast and self-adaptive FCM pulmonary nodule segmentation method combining clustering and classification learning is proposed in order to obtain satisfactory segmentations for blood vessel adhesion, pleura adhesion, and GGO pulmonary nodules. The new method improves the traditional FCM algorithm according to features of the above pulmonary nodules. It updates fuzzy memberships of central pixels based on grayscale similarities and spatial similarities of neighborhoods, so that clustering centers can be obtained quickly. The new method solves the problems of traditional segmentation methods such as the reliance on the contrast between objectives and backgrounds, difficulty to obtain weak edges of pulmonary nodules and attached tissues, slow convergence of objective functions, and so forth. In the meantime, according to the artificial labeled data, we can realize the aided supervision for pulmonary nodule segmentation missing classification information. Experimental results indicate that the newly proposed algorithm can segment blood vessel adhesion, pleura adhesion, and GGO pulmonary nodules fast and exactly and performs better than traditional FCM, FCM_S, EnFCM, FGFCM, and FLICM in segmentation effects, executive efficiencies, and rates of error.

However, there are still some disadvantages about the new method. For example, it does not work well for segmenting tiny nodules (diameter < 1 cm) and segmentations of GGO nodules and tightly adhered nodules are unstable. In future research, we will solve these problems by extracting more characteristics of pulmonary nodules and deep learning method. We will also analyze the symptoms of benign and malignant nodules comprehensively and provide real aided diagnosis for early detection and screening of lung cancer.

## Figures and Tables

**Table:** (a) Central pixel *x*_*j*_ is the noise of tiny blood vessel in Figure 7(a)

Greyscale			Initial membership		
78	53	53	0.22	0.11	0.39
60	142	52	0.82	0.13	0.25
60	59	56	0.42	0.54	0.25

**Table:** (b) Central pixel *x*_*j*_ is connection of nodule and lung wall in Figure 7(b)

Greyscale			Initial membership		
132	142	162	0.27	0.89	0.78
140	135	168	0.55	0.18	0.44
153	156	138	0.54	0.84	0.42

**Table:** (a) After 1 iteration

0.4753		0.4727		0.4712
0.4717		0.4723		0.4753
0.4721		0.4736		0.4722
	*v*_1_ = 80.54		*v*_2_ = 37.46	

0.5031		0.5122		0.5124
0.5112		0.53031		0.5042
0.5121		0.5063		0.5066
	*v*_1_ = 83.55		*v*_2_ = 42.68	

**Table:** (b) After 4 iterations

0.7564		0.7562		0.7601
0.7537		0.7523		0.7564
0.8120		0.7611		0.7589
	*v*_1_ = 52.44		*v*_2_ = 63.27	

0.7781		0.7881		0.7689
0.7833		0.7682		0.7596
0.7681		0.8195		0.7764
	*v*_1_ = 36.88		*v*_2_ = 81.67	

**Table:** (c) After 7 iterations

0.9687		0.9311		0.9714
0.9658		0.9602		0.9596
0.9709		0.9673		0.9598
	*v*_1_ = 20.74		*v*_2_ = 98.65	

0.9868		0.9727		0.9943
0.9907		0.9739		0.9763
0.9960		0.9902		0.9856
	*v*_1_ = 7.29		*v*_2_ = 118.69	

**Table:** (a) After 1 iteration

0.6007		0.5507		0.5677
0.5236		0.5712		0.5178
0.5807		0.5008		0.5438
	*v*_1_ = 82.25		*v*_2_ = 30.58	

0.4922		0.4993		0.4892
0.5934		0.4993		0.5372
0.4993		0.4898		0.5192
	*v*_1_ = 71.45		*v*_2_ = 44.86	

**Table:** (b) After 4 iterations

0.3446		0.3247		0.3251
0.4024		0.3512		0.3415
0.3515		0.3861		0.3488
	*v*_1_ = 46.28		*v*_2_ = 76.55	

0.2452		0.3412		0.2944
0.3544		0.2586		0.3589
0.2881		0.3245		0.2292
	*v*_1_ = 34.62		*v*_2_ = 82.54	

**Table:** (c) After 7 iterations

0.0278		0.0852		0.0353
0.0267		0.0497		0.0318
0.0795		0.0278		0.0519
	*v*_1_ = 20.48		*v*_2_ = 100.56	

0.0105		0.0084		0.0068
0.0097		0.0092		0.0087
0.0082		0.0081		0.0051
	*v*_1_ = 5.33		*v*_2_ = 118.36	

**Table 4:** Databases used in the experiment.

	LIDC	Qianfoshan Hospital, Shandong Province
Average number of sections in each set	100	150
Number of 2D CT images for experiment	48	67
Image resolution	512 × 512	512 × 512
Thickness of sections	2~3 mm	1~3 mm
Diameter of pulmonary nodules	8–30 mm	7–30 mm
Number of pulmonary nodules		
Isolated	64	98
Blood vessel adhesion	41	56
Pleural adhesion	19	39
ground glass opacity	25	21

**Table 5:** Comparison of executive efficiencies of the six algorithms.

Algorithm	Number of iterations			
	Legend			
	Figure 5 (noise added)	Figure 6	Figure 7	Figure 8
FCM	69	50	35	68
FCM_S	36	14	22	63
EnFCM	11	28	13	56
FGFCM	11	27	14	47
FLICM	18	33	13	15
Algorithm in this paper	6	8	5	9

	Running time (s)			

FCM	12.8605	12.4230	9.8213	17.2120
FCM_S	10.9752	9.1020	8.3464	14.3180
EnFCM	7.2450	8.3830	8.1840	12.3040
FGFCM	9.2880	7.4350	6.1859	9.3020
FLICM	6.2450	9.3020	6.1826	7.7290
Algorithm in this paper	5.3614	4.6481	4.4618	5.7344
